# Antinociceptive Effect and Hyperalgesia of Fentanyl and Its Analogues

**DOI:** 10.3390/ijms27073028

**Published:** 2026-03-26

**Authors:** Yuanyuan Chen, Kaixi Li, Xiangyu Li, Simeng Zhang, Deli Xu, Yawen Xu, Yanling Qiao, Yizhao Xu, Mengchan Xia, Weitao Qin, Bin Di, Peng Xu

**Affiliations:** 1 School of Pharmacy, China Pharmaceutical University, Nanjing 210009, China; 2 Office of China National Narcotics Control Commission, China Pharmaceutical University Joint Laboratory on Key Technologies of Narcotics Control, Beijing 100193, China; 3 Key Laboratory of Drug Monitoring and Control, Drug Intelligence and Forensic Center, Ministry of Public Security, Beijing 100193, China; 4 National Narcotics Laboratory Beijing Regional Center, Beijing 100164, China; 5 School of Investigation, People’s Public Security University of China, Beijing 100038, China

**Keywords:** opioid, fentanyl, analogue, antinociception, hyperalgesia, hot plate test, mice

## Abstract

Fentanyl is a potent analgesic widely used in clinical practice. Fentanyl and its analogues are seriously abused and are emerging in the illegal drug market, leading to numerous intoxication cases. However, assessment of the potency of the pharmacological effect of these novel fentanyl analogues remains limited and inconsistent across studies. The development of novel analgesics has largely relied on the assessment of mu opioid receptor (MOR) binding affinity, with insufficient verification through the assessment of antinociceptive effects. This study evaluated the antinociceptive effects of 25 fentanyl analogues to investigate the relationship between chemical structure and antinociceptive effect. In this study, hot plate tests were conducted in mice to generate time–effect and dose–effect curves for the evaluation of the antinociceptive effect of fentanyl and its analogues. The results demonstrated that the antinociceptive effects of fentanyl analogues were dose- and time-dependent. The potency of the antinociceptive effect observed in this study generally aligned with the corresponding MOR binding affinities reported in the literature, although several analogues exhibited discrepancies. Structural modifications in different regions of the fentanyl scaffold affect the antinociceptive potency to different degrees, and the duration of action also varied across fentanyl analogues. Furthermore, opioid-induced hyperalgesia (OIH) was observed following administration of several fentanyl analogues, raising potential concerns regarding their abuse liability and development for analgesic purposes. Taken together, this study systematically evaluated and compared the antinociceptive effects of fentanyl analogues. The findings clarify the relationship between chemical structure and the antinociceptive effect, providing valuable insights for drug regulation and the development of novel analgesics.

## 1. Introduction

Fentanyl and its analogues have increasingly been associated with numerous cases of intoxication and overdose fatalities. They are considered the leading cause of drug-related overdose deaths among adults aged 18–45 in North America [1]. As a class of new psychoactive substances (NPS), fentanyl and its analogues can be readily synthesized, facilitating their illicit production [1]. Structural modifications are often introduced to the fentanyl scaffold to circumvent drug controls. To date, fourteen fentanyl analogues have been placed under Schedule I and/or Schedule IV of the Single Convention on Narcotic Drugs of 1961. Over 80 fentanyl analogues have been reported to the United Nations Office on Drugs and Crime (UNODC), and more than 1400 fentanyl analogues have been described in the literature [2]. In response to this public health challenge, China has placed the entire class of fentanyl analogues under control in 2019.

**Figure 1:**
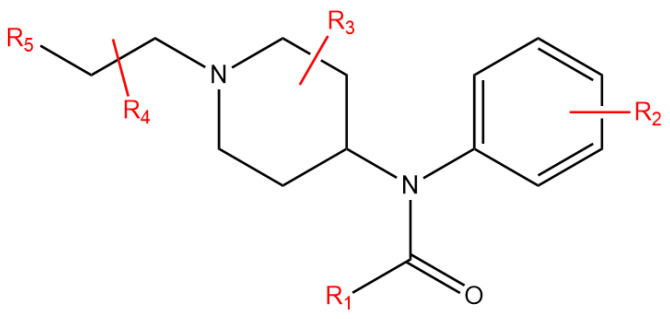
Modifications to the chemical structure of fentanyl scaffold.

Fentanyl is a potent analgesic widely used in clinical practice. Its antinociceptive potency is reported to be 50–100 times greater than that of morphine. Three fentanyl analogues—alfentanil, remifentanil, and sufentanil—have also been approved for medical use. Carfentanil is an ultra-potent analogue and has been approved for the anaesthetization of large animals [3]. Numerous fentanyl analogues have been derived from fentanyl and investigated for potential pharmaceutical applications, though they have not been commercialized (Figure 1). The side effects of fentanyl and its analogues, including respiratory depression and addiction liability, have raised significant public concerns regarding their misuse and abuse. In recent years, as fundamental research on analgesia has advanced, renewed interest has emerged in modifying the fentanyl scaffold to develop safer and more effective analgesics. Different pharmacological effects may be attributed to biassed signalling pathways of the mu opioid receptor (MOR) [4,5]. The relationship between the chemical structure and the antinociceptive and side effects of fentanyl and its analogues is under investigation. However, current data remain largely limited to MOR binding affinity. Importantly, the binding affinity does not always correlate with the in vivo potency of various pharmacological effects. Furthermore, studies evaluating the antinociceptive effect of fentanyl analogues have employed diverse animal models and experimental protocols, limiting direct comparability across findings. Therefore, further research on the antinociceptive effect of fentanyl analogues is warranted to clarify the relationship between chemical structure and antinociceptive effect.

The antinociceptive effect can be evaluated via various animal models. Among these, the hot plate test, tail withdrawal test, acetic acid-induced writhing test and formalin-induced paw test are the most widely used methods [6]. The hot plate test is a typical rodent model involving supraspinal sensory processing [7]. Fentanyl and its analogues are small lipophilic molecules that are capable of rapidly crossing the blood–brain barrier (BBB) to exert effects on the central nervous system (CNS). Therefore, we employed the hot plate test to evaluate the antinociceptive effect of 25 fentanyl analogues (Table 1). Additionally, data from four other fentanyl analogues—previously evaluated and published in earlier studies from our laboratory or currently under review in other publications—were included in the analysis. The aim of this study was to provide comparative information on in vivo antinociceptive potency and structure–effect trends of fentanyl analogues.

## 2. Results

**Figure 2:**
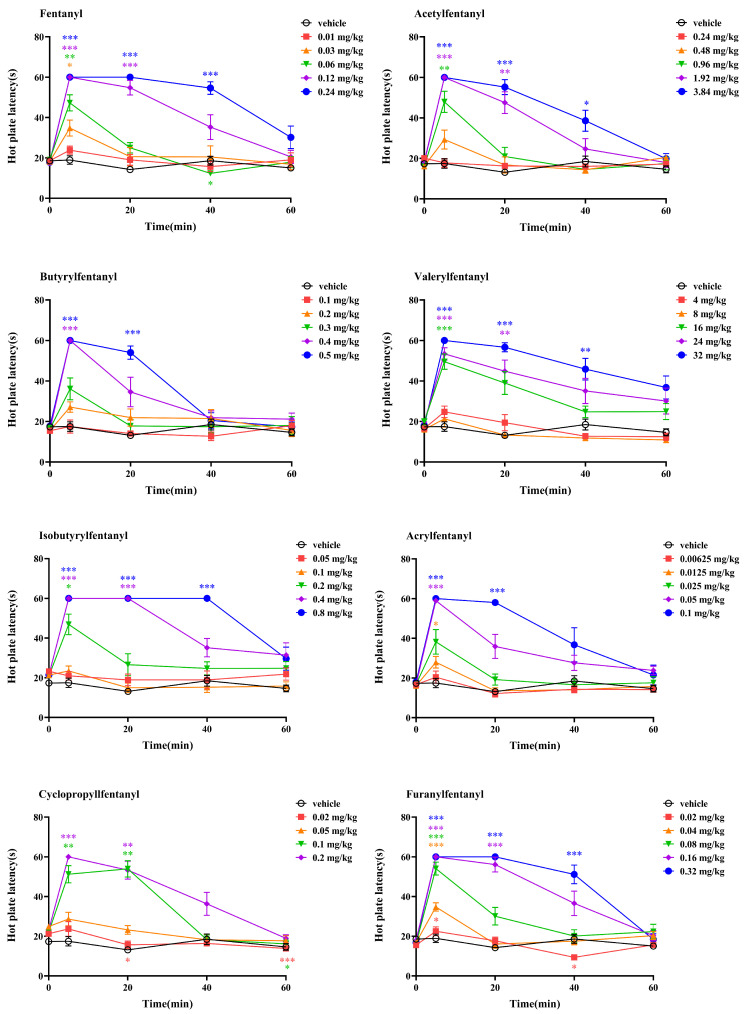

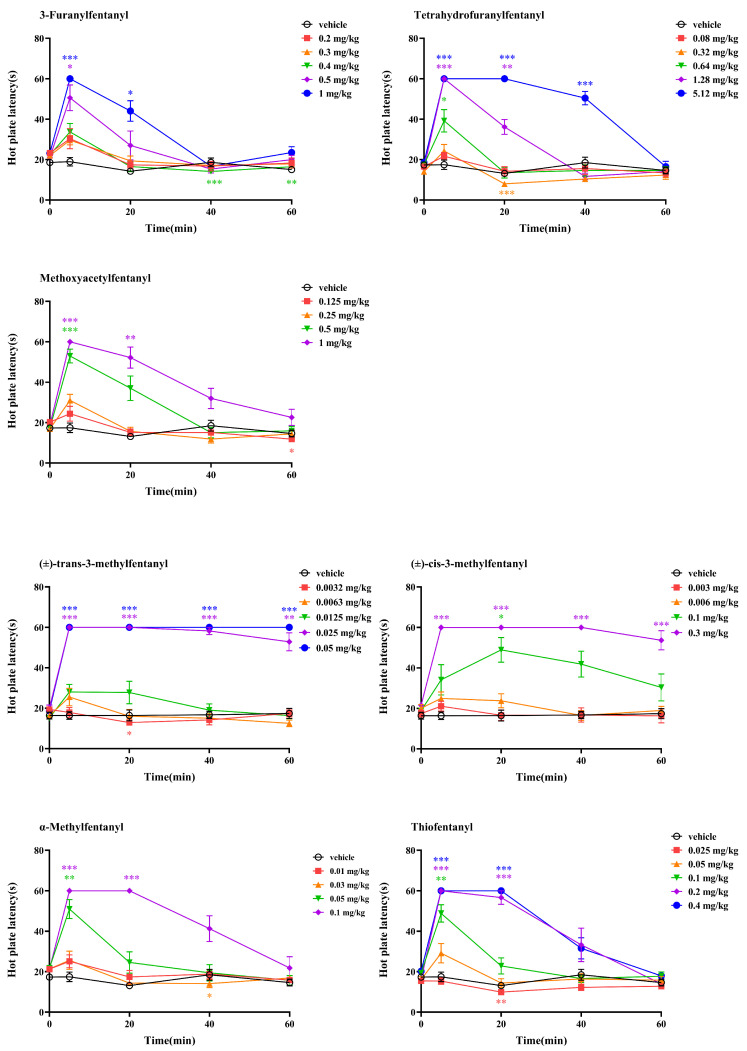

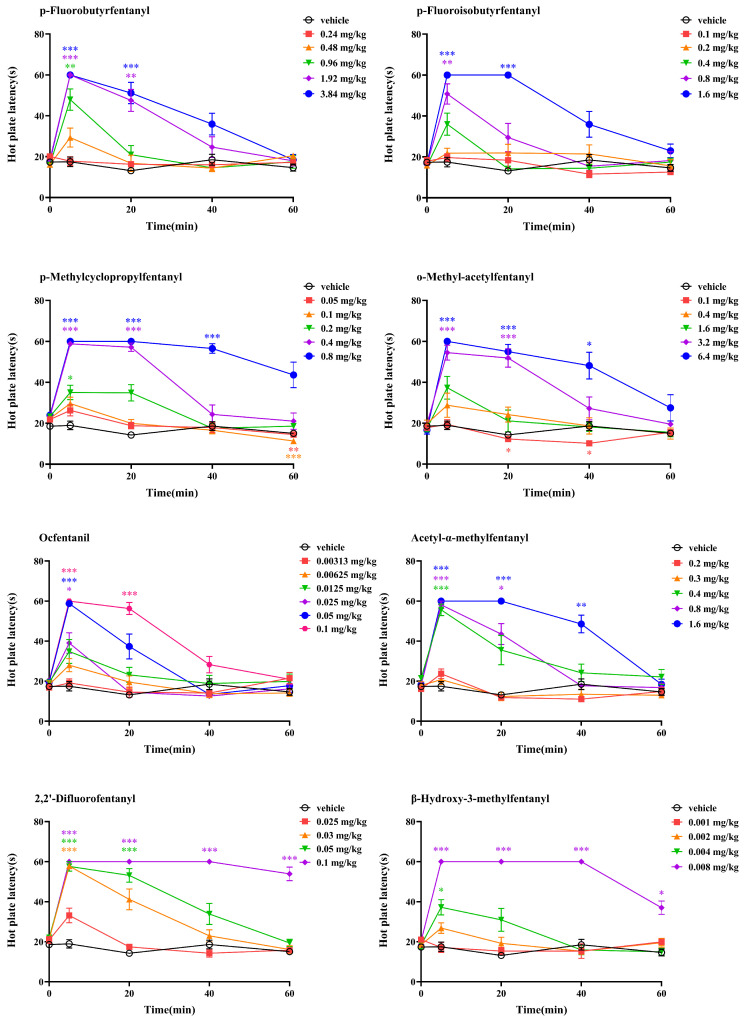

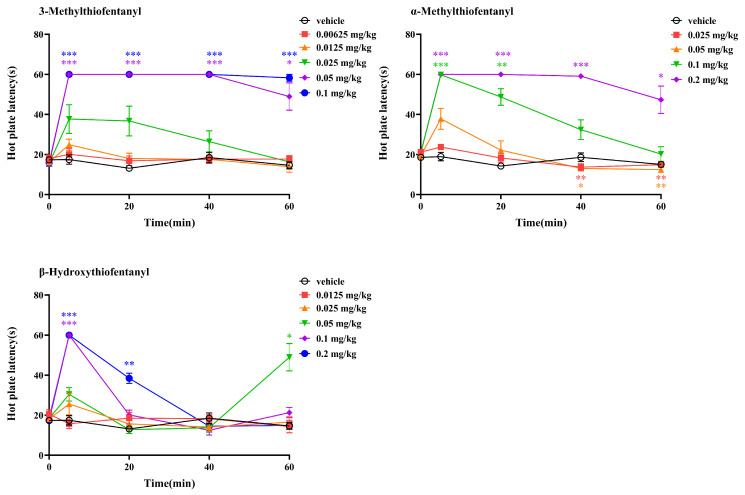
Time–effect curves for the antinociceptive effect of fentanyl analogues. All data are presented as mean ± SEM for *n* = 7–11 mice per group. Two-way ANOVA, Bonferroni post hoc test, * *p* < 0.05, ** *p* < 0.01, *** *p* < 0.001, compared with the control latency for each group.

**Figure 3:**
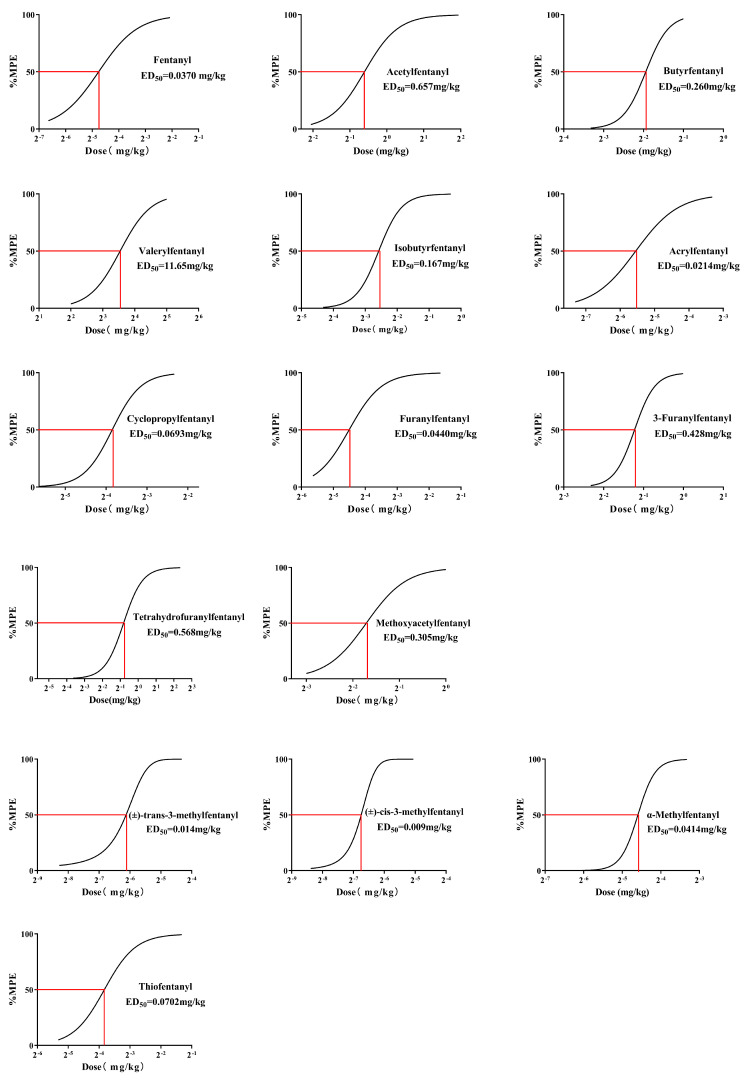

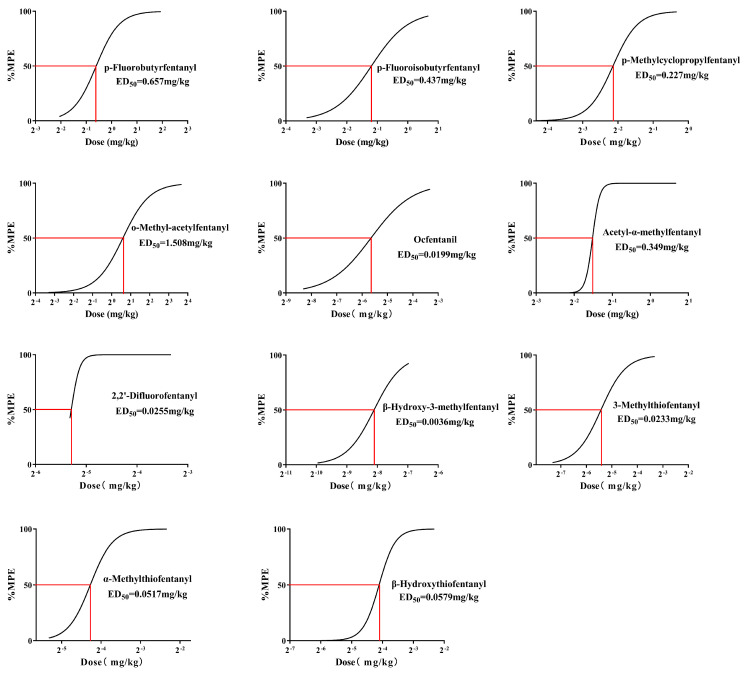
Dose–effect curves for the antinociceptive effect and ED_50_ values of fentanyl analogues. The percent maximum possible effect (%MPE) was calculated from the pain threshold measured 5 min after drug administration. All the data are presented as mean ± SEM for *n* = 7–11 mice per group.

The antinociceptive effect of 25 fentanyl analogues was evaluated via the hot plate test, with fentanyl as the reference. The results are shown in Figure 2 and Figure 3. All fentanyl analogues elicited the maximum antinociceptive effect 5 min after administration (Figure 2). Two-way ANOVA revealed that fentanyl analogues produced an antinociceptive effect in a dose- and time-dependent manner. F and P values are presented in Table 2.

Like fentanyl, the antinociceptive effect of most fentanyl analogues was still significant 20 min after administration at the dose at which hot plate latency first reached 60 s. However, for butyrylfentanyl, acrylfentanyl, ocfentanil and β-hydroxythiofentanyl, the antinociceptive effect lasted less than 20 min. For valerylfentanyl and o-methyl-acetylfentanyl, the antinociceptive effect lasted for at least 40 min. For (±)-trans-3-methylfentanyl, (±)-cis-3-methylfentanyl, β-hydroxy-3-methylfentanyl and 3-methylthiofentanyl, the antinociceptive effect lasted for at least 60 min. Furthermore, the pain threshold decreased below the baseline for several fentanyl analogues after administration at small doses. The pain threshold decreased 20 min and 60 min after administration of 0.02 mg/kg, and 60 min after administration of 0.1 mg/kg for Cyclopropylfentanyl, 40 min after administration of 0.02 mg/kg for furanylfentanyl, 40 to 60 min after administration of 0.4 mg/kg for 3-furanylfentanyl, 20 min after administration of 0.32 mg/kg for tetrahydrofuranylfentanyl, 60 min after administration of 0.125 mg/kg for methoxyacetylfentanyl, 20 min after administration of 0.0032 mg/kg for (±)-trans-3-methylfentanyl, 40 min after administration of 0.03 mg/kg for α-Methylfentanyl, 20 min after administration of 0.025 mg/kg for thiofentanyl, 60 min after administration of 0.05 and 0.1 mg/kg for p-Methylcyclopropylfentanyl, 20 to 40 min after administration of 0.1 mg/kg for o-Methyl-acetylfentanyl, and 40 to 60 min after administration of 0.025 and 0.05 mg/kg for α-Methylthiofentanyl. This phenomenon was also observed with fentanyl. The pain threshold decreased 40 min after administration of 0.06 mg/kg of fentanyl. In addition, the hot plate latency rebounded significantly 60 min after administration of 0.05 mg/kg for β-Hydroxythiofentanyl, which was uncommon.

The ED_50_ values of the hot plate test were achieved by analysing the relationship between the dose and the maximum antinociceptive response 5 min after administration. The curves between dose and %MPE were S-shaped (Figure 3). ED_50_ values varied among differently modified fentanyl analogues.

## 3. Discussion

Fentanyl is a potent synthetic analgesic used clinically. Considerable efforts have been devoted to studying the relationship between the structure and pharmacological effect of the fentanyl scaffold to develop potent and safer analgesics. However, data on fentanyl analogues vary considerably across studies, limiting direct comparability. Moreover, previous research has shown that the antinociceptive effects of fentanyl analogues do not always correlate with their MOR binding affinity [11]. To better elucidate the relationship between structure and antinociceptive effect, this study evaluated the antinociceptive effect of 25 fentanyl analogues under a consistent experimental protocol. Data from the literature regarding MOR binding affinity and antinociceptive effect of fentanyl analogues, together with the data obtained in this study, are summarized in Table 3. Accordingly, there were three main findings. First, the potency of the antinociceptive effect generally correlated with MOR binding affinity. Second, the relationship between chemical structure and pharmacological effect was further clarified through standardized evaluation. Third, opioid-induced hyperalgesia (OIH) was frequently observed among fentanyl analogues even after acute administration.

Data obtained in the hot plate test are generally consistent with those reported in previous studies and are summarized in Table 3, except for carfentanil. However, the potency of the antinociceptive effect showed limited correlation between the hot plate test and the tail withdrawal test. For example, isobutyrfentanyl exhibited an antinociceptive potency that was 0.2-fold lower than fentanyl in the hot plate test, whereas it demonstrated comparable potency to fentanyl in the tail withdrawal test. Discrepancies were also observed for acrylfentanyl, p-fluorofentanyl, and β-hydroxythiofentanyl. Notably, data from the hot plate test remain relatively scarce in the literature compared to those from the tail withdrawal test. Moreover, results from the tail withdrawal test vary across studies, likely due to differences in experimental protocols and animal species used. The hot plate test involves supraspinal sensory processing, whereas the tail withdrawal test primarily measures a simple rodent withdrawal reflex [7]. The mechanism of the antinociceptive effect of opioids involves their effects on brain regions, the spinal cord and the peripheral nervous system [28,29]. Pain is defined as an unpleasant sensory or emotional state, with the CNS playing a complex and integral role in its modulation [11]. The observed discrepancies between the two models may result from the differences in CNS engagement and the BBB permeability of the tested fentanyl analogues. Stress, learning, sedation, and several other factors may also contribute to the discrepancies.

Like other opioids, fentanyl preferentially binds to MOR to produce pharmacological effects such as antinociceptive effects and respiratory depression. Previous studies have shown that, for several fentanyl analogues, antinociceptive potency does not consistently correlate with MOR binding affinity [11,27]. In contrast, our findings indicate that the antinociceptive potency of the fentanyl analogues evaluated in this study generally aligns with their reported MOR binding affinity. Nevertheless, certain analogues deviate from this trend. The potency of cyclopropylfentanyl in MOR binding affinity varied among different studies. For instance, cyclopropylfentanyl exhibited an antinociceptive effect that was 0.5-fold lower than that of fentanyl in the hot plate test, despite previous reports indicating comparable potency and efficacy in both MOR binding and antinociceptive activity [16]. Discrepancies in the potency of cyclopropylfentanyl across studies may be attributed to variations in experimental protocols and animal species used. Furanylfentanyl demonstrates antinociceptive potency similar to that of fentanyl, consistent with prior findings, even though it displayed higher MOR binding affinity [27]. Acrylfentanyl was found to be 1.7 times more potent than fentanyl in antinociceptive effects, while its MOR binding affinity was reported to be comparable to fentanyl in two independent studies (Table 3). Several factors may contribute to the inconsistency between the potency of the antinociceptive effect and MOR binding affinity. MOR binding kinetics can be further studied in combination with binding affinity. Furthermore, MOR binding affinities were often assessed in vitro. The pharmacological effect in vivo is more complex. BBB permeability is an important factor since opioids mainly produce antinociceptive effects by binding to MOR in CNS, and P-glycoprotein also plays a vital role in BBB permeability [30]. The pharmacokinetic parameters of fentanyl analogues also matter since they influence the drug distribution and concentration in the brain [31].

The relationship between the chemical structure and pharmacological effect of fentanyl analogues has been extensively studied [11]. Structural modifications in different regions of the fentanyl scaffold structure can lead to significant variations in pharmacological potency. Among analogues modified at the N-propionyl group, ethyl substitution on the propionyl moiety yields the greatest antinociceptive potency. Both longer and shorter alkyl chains resulted in reduced antinociceptive potency. The decreased potency of methoxyacetylfentanyl suggested that insertion of an oxygen atom into the propionyl moiety diminishes the potency of fentanyl. Acrylfentanyl exhibited greater antinociceptive potency than fentanyl. Furanylfentanyl demonstrates comparable potency. 3-Furanylfentanyl and tetrahydrofuranylfentanyl showed reduced antinociceptive potency. For analogues modified on the piperidine ring of the fentanyl scaffold, the high potency of 3-methylfentanyl and carfentanil indicated that 3-methyl and 4-carbomethoxy substitution significantly enhanced the antinociceptive effect. Regarding modifications on the aniline ring, fluorine substitution at the ortho position increases antinociceptive potency relative to fentanyl, while para-substitution reduces it. Results for p-Fluorobutyrfentanyl and p-Fluoroisobutyrfentanyl further verify this hypothesis. However, methyl substitution at either the ortho or para position of the aniline ring led to decreased potency. In analogues modified on the phenethyl moiety, α-Methylfentanyl displayed potency similar to that of fentanyl. The potency of thiofentanyl decreased. Results of isobutyryl-carfentanyl and acetyl-α-methylfentanyl confirmed that isopropyl and methyl substitution on the propionyl moiety were associated with lower potency. 2′-fluorine substitution on the aniline ring decreased the potency. Modification involving multiple regions of the fentanyl scaffold generally aligned with the finding observed for substitution in one region for most fentanyl analogues. However, notable exceptions exist: the high potency of ocfentanil demonstrated that methyoxymethyl substitution on the propionyl region and ortho-methyl substitution on the aniline region can synergistically enhance the antinociceptive effect. Furthermore, β-hydroxy substitution on the phenethyl region increased the potency of 3-methylfentanyl but had minimal impact on thiofentanyl. Such enhancement by β-hydroxy substitution has been previously reported in the literature [11]. Overall, the majority of fentanyl analogues exhibit higher antinociceptive potency compared to morphine.

Furthermore, we observed that the duration of the antinociceptive effect varied among fentanyl analogues with different structural modifications. For fentanyl and most of its analogues, the antinociceptive effect persisted for 20 min after administration. For (±)-trans-3-methylfentanyl, (±)-cis-3-methylfentanyl, β-hydroxy-3-methylfentanyl, and 3-methylthiofentanyl, the antinociceptive effect lasted at least 60 min after administration. The insertion of the 3-methyl group into the fentanyl scaffold appeared to prolong the duration of the antinociceptive effect. This is consistent with findings for lofentanil, a derivative of carfentanil featuring a 3-methyl substitution, which has been reported to have a significantly prolonged duration of action in the literature [11]. 3-Methylfentanyl has been reported to induce wash-resistant inhibition of MOR [32]. Conversely, butyrylfentanyl, acrylfentanyl, ocfentanil and β-hydroxythiofentanyl produced antinociceptive effects that lasted less than 20 min after administration. The short duration of ocfentanil has been previously documented, and our results are in agreement with prior reports [27]. Overall, the duration of antinociceptive effects may be influenced by multiple factors, including MOR binding properties and pharmacokinetic parameters [33,34].

In this study, we found an interesting phenomenon called opioid-induced hyperalgesia (OIH). Our study demonstrated that acute administration of fentanyl and 11 fentanyl analogues, cyclopropylfentanyl, furanylfentanyl, 3-furanylfentanyl, tetrahydrofuranylfentanyl, methoxyacetylfentanyl, (±)-trans-3-methylfentanyl, α-methylfentanyl, thiofentanyl, p-methylcyclopropylfentanyl, o-methyl-acetylfentanyl and α-methylthiofentanyl, induced OIH in the hot plate test. OIH is commonly encountered in opioid administration when used clinically, including agents such as fentanyl, remifentanil, and morphine. It poses a significant clinical challenge [35,36,37,38]. OIH often occurs after prolonged use of opioids and at doses exceeding those required for analgesia [39,40,41]. Waxman et al. [42] demonstrated that both an acute bolus fentanyl dose (0.25 mg/kg, s.c.) and continuous infusion of fentanyl (cumulative daily dose: 10 mg/kg) can induce OIH in mice. OIH is also studied at subanalgesic and analgesic doses [43]. The underlying mechanism contributing to OIH has not been fully clarified. The literature suggests that the mechanism of OIH may not depend on MOR, but rather exhibit greater correlation with NMDA receptors [42]. However, recent research has indicated a close relationship between MOR and the mechanisms underlying OIH, and both the brain and spinal system were involved [44]. OIH is also studied via the biassed pathway of MOR, as with other side effects [43]. Pain is defined as an unpleasant sensory or emotional state [11]. Koob et al. posited potential connections between pain (hyperalgesia) and negative emotional states (hyperkatifeia), which were both unpleasant states after opioid use [39,45]. Research has shown that prolonged opioid usage shifts the central process of pain from sensory to emotional and cognitive brain areas and may produce a tendency towards pain catastrophizing characterized by exaggerating, focusing on, and feeling helpless about the pain experience [46]. Strong evidence suggests that neuroadaptation of the stress system associated with addiction may overlap with substrates of emotional aspects of pain processing in areas such as the amygdala [39,47]. Previous studies have indicated that OIH reflects an unstable state of the body and can enhance the negative reinforcing effect of opioids, thereby contributing to relapse [39,46,48]. The addictive property of fentanyl and other opioids is a clinical concern. It is crucial to consider OIH when studying the addictive properties of fentanyl analogues and developing novel analgesics. In addition, the evaluation of OIH induced by continuous administration of fentanyl and its analogues could be conducted in conjunction with the negative reinforcing effect and relapse. Notably, although the negative control group did not show hot plate latency below baseline, the possibility of habituation and learning to lick the hind paw should also be taken into consideration. Stress sensitization and motor or sedation rebound may also contribute to this. More studies should be conducted to confirm the phenomenon of OIH. The MOR mediation can be further confirmed by the naloxone reversal test.

Our study has certain limitations. Only female mice were used in the experiment. Sex and gender play important roles in analgesic effects. In pain and opioid analgesia, the difference between males and females is complex and controversial [49]. Clinical studies have shown that the pain threshold of females is lower than that of males. Women are more sensitive to pain and have a lower pain tolerance. Preclinical studies have shown that although there have been various studies on the difference in pain sensitivity between males and females, the results have varied across studies [49]. Some studies have shown a notable sex difference in pain sensitivity, whereas other studies have not. Furthermore, sex differences in response to opioid analgesics have been studied. In clinical studies, women were reported to be more sensitive to opioid analgesics and to consume lower amounts of opioids than men for acute pain relief [50,51]. The pharmacokinetics of opioids also differ between males and females [52]. Gonadal hormones influence both pain sensitivity and the analgesic effect of opioids. Testosterone was reported to increase the pain threshold, and estrogen fluctuations were reported to increase pain intensity and perception [53]. Oestrogen was reported to modulate opioid analgesia negatively, while progestin was reported to promote opioid analgesia and increase the expression of opioid receptors [54]. The influence of sex and gender on fentanyl analgesia and antinociception can be further investigated as an interesting aspect.

## 4. Materials and Methods

### 4.1. Drugs

Fentanyl hydrochloride, acetylfentanyl, butyrfentanyl, valerylfentanyl, isobutyrfentanyl, acrylfentanyl, p-fluorobutyrfentanyl, p-fluoroisobutyrfentanyl, cyclopropylfentanyl, p-methylcyclopropylfentanyl, furanylfentanyl, 3-furanylfentanyl, tetrahydrofuranylfentanyl hydrochloride, α-methylfentanyl oxalate, acetyl-α-methylfentanyl, β-hydroxy-3-methylfentanyl, o-methyl acetylfentanyl, methoxyacetylfentanyl hydrochloride, 2,2′-difluorofentanyl, ocfentanil, thiofentanyl hydrochloride, 3-methylthiofentanyl hydrochloride, α-methylthiofentanyl and β-hydroxythiofentanyl hydrochloride were supplied by the Drug Intelligence and Forensic Centre of the Ministry of Public Security (Beijing, China). All drugs were dissolved in either sterile saline or citrate buffer solution, which have been described in previous studies [9]. The final solution for all drugs used for administration was prepared with 0.9% saline.

### 4.2. Subjects

Female ICR mice weighing 18–22 g and aged 6–8 weeks were obtained from Beijing Sipeifu Biotechnology Co., Ltd. (Beijing, China). Subjects were housed in a temperature- and humidity-controlled environment (temperature: 25 ± 2 °C, humidity: 60 ± 10%) and maintained on a 12/12 h light/dark cycle. Food and water were available ad libitum.

Animals were acclimated for at least 7 days before any experimental manipulation. To minimize stress during behavioural testing, animals were handled daily for 3 days prior to the start of experiments and habituated to the testing room for 30 min before each test. Animals showing signs of severe distress (e.g., weight loss > 15%, inability to ambulate, hunched posture, persistent piloerection) were immediately euthanized. Upon completion of the experiment, animals were euthanized by carbon dioxide inhalation.

### 4.3. Hot Plate Test

The test was conducted using a hot plate analgesia metre (Beijing Zhongshidichuang Science and Technology Development Co., Ltd., Beijing, China). Female mice were used because the genitals of male mice may be scalded by the hot plate. Procedures were conducted as previously described [9]. Mice were placed on the plate, which was preheated to 55 ± 0.5 °C, and the time latency to the first sign of licking the hind paw was measured. Control latency was measured twice before drug treatment to select qualified mice with a latency between 5 s and 30 s, and mice that were too sensitive (latency < 5 s) or insensitive (latency > 30 s) were eliminated. A total of about 2500 mice were used, and about one-third of the mice were eliminated. The pain threshold was defined as the time latency from the mice being placed on the hot plate to the first sign of licking the hind paw.

Separated groups of qualified mice (*n* = 7–11 for each administration dose, s.c.) were treated with fentanyl or its analogues. Several doses of one drug were prepared and coded with randomly assigned numbers by one researcher. The test was conducted by another researcher. Latencies of each dose group of mice were measured 5 min, 20 min, 40 min, and 60 min after treatment. A maximum exposure time was set at 60 s (cut-off time) to avoid tissue damage.

### 4.4. Data Analysis

Data are presented as mean ± standard error of the mean (SEM). GraphPad Prism 8.0 (Graphpad Software, Inc., San Diego, CA, USA) was used for statistical analysis. Data were analysed by two-way analysis of variance (ANOVA). Following two-way ANOVA, post hoc analysis was conducted using Bonferroni’s multiple comparisons test, and a value of *p* < 0.05 was considered statistically significant.

The antinociceptive effect was quantified with the formula of the maximum possible effect (%MPE) calculated as [(latency after treatment − control latency)/(cut-off time − control latency) × 100%]. The analysis of the relationship between dose and the maximum antinociceptive response 5 min after administration was conducted, and the median effective dose (ED_50_) values of antinociception and 95% confidence interval (95% CI) were determined by nonlinear regression analysis.

## 5. Conclusions

Fentanyl is a potent synthetic analgesic. Fentanyl and its analogues have increasingly appeared in the illegal drug market and in intoxication cases, while simultaneously being investigated for the development of potent and safe analgesics. This study systematically evaluated and compared the antinociceptive effect of fentanyl analogues. The findings highlight the structure–effect relationship and provide valuable insights for drug regulation and the development of novel analgesic agents.

## Figures and Tables

**Table 1:** Different modifications to fentanyl scaffold structure of fentanyl analogues.

	Name	R1	R2	R3	R4	R5
1	Fentanyl	ethyl	H	H	H	phenyl
Modification on R1						
2	Acetylfentanyl	methyl	H	H	H	phenyl
3	Butyrfentanyl	propyl	H	H	H	phenyl
4	Valerylfentanyl	butyl	H	H	H	phenyl
5	Isobutyrfentanyl	isopropyl	H	H	H	phenyl
6	Acrylfentanyl	ethylene	H	H	H	phenyl
7	Cyclopropylfentanyl	cyclopropyl	H	H	H	phenyl
8	Furanylfentanyl	2-furanyl	H	H	H	phenyl
9	3-Furanylfentanyl	3-furanyl	H	H	H	phenyl
10	Tetrahydrofuranylfentanyl	2-tetrahydrofuranyl	H	H	H	phenyl
11	Methoxyacetylfentanyl	methyoxymethyl	H	H	H	phenyl
Modification on R2						
12	p-Fluorofentanyl *	ethyl	p-fluoro	H	H	phenyl
13	o-Fluorofentanyl *	ethyl	o-fluoro	H	H	phenyl
Modification on R3						
14	(±)-trans-3-Methylfentanyl	ethyl	H	trans-3-methyl	H	phenyl
15	(±)-cis-3-Methylfentanyl	ethyl	H	cis-3-methyl	H	phenyl
16	Carfentanil *	ethyl	H	4-carbomethoxy	H	phenyl
Modification on R4						
17	α-Methylfentanyl	ethyl	H	H	α-methyl	phenyl
Modification on R5						
18	Thiofentanyl	ethyl	H	H	H	2-thiophene
Modifications on more than one region						
19	p-Fluorobutyrfentanyl	propyl	p-fluoro	H	H	phenyl
20	p-Fluoroisobutyrfentanyl	isopropyl	p-fluoro	H	H	phenyl
21	p-Methylcyclopropylfentanyl	cyclopropyl	p-methyl	H	H	phenyl
22	o-Methyl-acetylfentanyl	methyl	o-methyl	H	H	phenyl
23	Ocfentanil	methyoxymethyl	o-methyl	H	H	phenyl
24	Isobutyryl-carfentanyl *	isopropyl	H	4-carbomethoxy	H	phenyl
25	Acetyl-α-methylfentanyl	methyl	H	H	α-methyl	phenyl
26	2,2′-Difluorofentanyl	ethyl	o-fluoro	H	H	o-fluorophenyl
27	β-Hydroxy-3-methylfentanyl	ethyl	H	3-methyl	β-hydroxy	phenyl
28	3-Methylthiofentanyl	ethyl	H	3-methyl	H	2-thiophene
29	α-Methylthiofentanyl	ethyl	H	H	α-methyl	2-thiophene
30	β-Hydroxythiofentanyl	ethyl	H	H	β-hydroxy	2-thiophene

* meant the data of the drug in our lab has been published [8,9,10].

**Table 2:** F and *p* values of time–effect curves for the antinociceptive effect of fentanyl analogues.

		Dose		Time		Dose × Time Interaction	
		F	*p*	F	*p*	F	*p*
1	Fentanyl	F(5, 56) = 32.89	*p* < 0.0001	F(3.451, 193.3) = 63.54	*p* < 0.0001	F(20, 224) = 20.00	*p* < 0.0001
2	Acetylfentanyl	F(5, 44) = 28.46	*p* < 0.0001	F(3.116, 137.1) = 65.50	*p* < 0.0001	F(20, 176) = 13.87	*p* < 0.0001
3	Butyrfentanyl	F(5, 45) = 16.04	*p* < 0.0001	F(3.330, 149.8) = 54.97	*p* < 0.0001	F(20, 180) = 11.43	*p* < 0.0001
4	Valerylfentanyl	F(5, 57) = 27.70	*p* < 0.0001	F(3.281, 187.0) = 45.43	*p* < 0.0001	F(20, 228) = 6.897	*p* < 0.0001
5	Isobutyrfentanyl	F(5, 44) = 52.64	*p* < 0.0001	F(3.013, 132.6) = 41.69	*p* < 0.0001	F(20, 176) = 14.52	*p* < 0.0001
6	Acrylfentanyl	F(5, 42) = 32.74	*p* < 0.0001	F(2.992, 125.7) = 49.88	*p* < 0.0001	F(20, 168) = 9.576	*p* < 0.0001
7	Cyclopropylfentanyl	F(4, 37) = 37.31	*p* < 0.0001	F(2.766, 102.3) = 51.75	*p* < 0.0001	F(16, 148) = 14.85	*p* < 0.0001
8	Furanylfentanyl	F(5, 55) = 57.81	*p* < 0.0001	F(3.043, 165.8) = 124.4	*p* < 0.0001	F(20, 218) = 21.96	*p* < 0.0001
9	3-Furanylfentanyl	F(5, 51) = 14.27	*p* < 0.0001	F(2.822, 143.9) = 62.99	*p* < 0.0001	F(20, 204) = 8.086	*p* < 0.0001
10	Tetrahydrofuranylfentanyl	F(5, 52) = 53.90	*p* < 0.0001	F(3.606, 187.5) = 125.0	*p* < 0.0001	F(20, 208) = 30.88	*p* < 0.0001
11	Methoxyacetylfentanyl	F(4, 40) = 28.86	*p* < 0.0001	F(2.862, 114.5) = 48.32	*p* < 0.0001	F(16, 160) = 10.29	*p* < 0.0001
12	(±)-trans-3-Methylfentanyl	F(5, 46) = 118.5	*p* < 0.0001	F(3.036, 138.9) = 55.21	*p* < 0.0001	F(20, 183) = 18.31	*p* < 0.0001
13	(±)-cis-3-Methylfentanyl	F(4, 35) = 42.69	*p* < 0.0001	F(3.167, 110.8) = 18.06	*p* < 0.0001	F(16, 140) = 7.621	*p* < 0.0001
14	α-Methylfentanyl	F(4, 35) = 31.23	*p* < 0.0001	F(3.418, 119.6) = 35.56	*p* < 0.0001	F(16, 140) = 12.16	*p* < 0.0001
15	Thiofentanyl	F(5, 46) = 39.20	*p* < 0.0001	F(2.650, 121.9) = 100.9	*p* < 0.0001	F(20, 184) = 22.05	*p* < 0.0001
16	p-Fluorobutyrfentanyl	F(5, 45) = 22.93	*p* < 0.0001	F(3.085, 138.8) = 64.13	*p* < 0.0001	F(20, 180) = 13.08	*p* < 0.0001
17	p-Fluoroisobutyrfentanyl	F(5, 42) = 23.28	*p* < 0.0001	F(3.492, 146.7) = 42.99	*p* < 0.0001	F(20, 168) = 10.34	*p* < 0.0001
18	p-Methylcyclopropylfentanyl	F(5, 54) = 71.30	*p* < 0.0001	F(2.956, 159.6) = 70.99	*p* < 0.0001	F(20, 216) = 16.70	*p* < 0.0001
19	o-Methyl-acetylfentanyl	F(5, 45) = 28.06	*p* < 0.0001	F(2.824, 126.4) = 46.20	*p* < 0.0001	F(20, 179) = 9.726	*p* < 0.0001
20	Ocfentanil	F(6, 53) = 18.79	*p* < 0.0001	F(3.415, 181.0) = 69.20	*p* < 0.0001	F(24, 212) = 10.47	*p* < 0.0001
21	Acetyl-α-methylfentanyl	F(5, 44) = 45.61	*p* < 0.0001	F(3.161, 139.1) = 95.32	*p* < 0.0001	F(20, 176) = 19.66	*p* < 0.0001
22	2,2′-Difluorofentanyl	F(4, 45) = 84.39	*p* < 0.0001	F(3.052, 137.3) = 114.4	*p* < 0.0001	F(16, 180) = 25.81	*p* < 0.0001
23	β-Hydroxy-3-methylfentanyl	F(4, 32) = 84.03	*p* < 0.0001	F(3.252, 104.1) = 24.91	*p* < 0.0001	F(16, 128) = 17.01	*p* < 0.0001
24	3-Methylthiofentanyl	F(5, 44) = 58.37	*p* < 0.0001	F(2.803, 120.5) = 67.56	*p* < 0.0001	F(20, 172) = 17.00	*p* < 0.0001
25	α-Methylthiofentanyl	F(4, 45) = 68.45	*p* < 0.0001	F(3.141, 141.3) = 58.62	*p* < 0.0001	F(16, 180) = 17.16	*p* < 0.0001
26	β-Hydroxythiofentanyl	F(5, 44) = 13.56	*p* < 0.0001	F(2.976, 130.9) = 52.69	*p* < 0.0001	F(20, 176) = 20.21	*p* < 0.0001

**Table 3:** Results compared with data published in the literature.

		Data Obtained in Our Lab			Data Published in the Literature					
		Hot Plate Test			Hot Plate Test		Tail Withdrawal Test		MOR Binding Affinity	
	Name	ED_50_ (mg/kg)	Potency Ratio to Morphine	Potency Ratio to Fentanyl	ED_50_ (mg/kg)	Potency Ratio to Fentanyl	ED_50_ (mg/kg)	Potency Ratio to Fentanyl	Ki (nM)	Potency Ratio to Fentanyl
	Morphine *	4.046	1.0	0.01	11.9 ^k^	0.003	7.82 ^o^	0.01	0.252 ^l^	0.5
1	Fentanyl	0.037	109.4	1.0	0.044 ^n^/0.026 ^k^	1.0	0.015 ^a^/0.08 ^b^/0.0091 ^c^/0.139 ^d^	1.0	0.135 ^l^/1.6 ^m^	1.0
Modification on R1										
2	Acetylfentanyl	0.657	6.2	0.06			0.28 ^c^	0.03	4.28 ^l^/64 ^m^	0.032/0.025
3	Butyrfentanyl	0.26	15.6	0.14			0.089 ^c^	0.1	0.405 ^l^/35 ^m^	0.33/0.05
4	Valerylfentanyl	11.65	0.3	0.003			6.43 ^b^	0.012	2.16 ^l^	0.063
5	Isobutyrfentanyl	0.167	24.2	0.2			0.0768 ^b^	1.04	0.291 ^l^/6.6 ^m^	0.46/0.24
6	Acrylfentanyl	0.0214	189.1	1.7			0.158 ^d^	0.9	0.133 ^l^/2.1 ^m^	1.0/0.8
7	Cyclopropylfentanyl	0.0693	58.4	0.5	0.048 ^p^		0.04 ^e^	0.75	0.088 ^l^/2.4 ^m^	1.5/0.67
8	Furanylfentanyl	0.044	92.0	0.8			0.02 ^f^	1	0.0279 ^l^/1.3 ^m^	4.8/1.2
9	3-Furanylfentanyl	0.428	9.5	0.09			0.51 ^g^	0.16	0.442 ^l^	0.31
10	Tetrahydrofuranylfentanyl	0.568	7.1	0.07			2.41 ^d^	0.06	0.95 ^l^/31 ^m^	0.14/0.052
11	Methoxyacetylfentanyl	0.305	13.3	0.12					17 ^m^	0.094
Modification on R2										
12	p-Fluorofentanyl *	0.088	46.0	0.4			0.06 ^o^	1.3	4.2 ^m^	0.38
13	o-Fluorofentanyl *	0.014	289.0	2.6			0.03 ^o^	2.7	0.4 ^m^	4
Modification on R3										
14	(±)-trans-3-Methylfentanyl	0.014	289.0	2.6	0.021 ^i^		0.0094 ^h^		1.1 ^m^	1.45
15	(±)-cis-3-Methylfentanyl	0.0098	412.9	3.8			0.0018 ^h^		0.32 ^m^	5
16	Carfentanil *	0.0026	1618.4	14.8	0.00041 ^i^				0.024 ^i^	
Modification on R4										
17	α-Methylfentanyl	0.0414	97.7	0.9			0.0085 ^h^			
Modification on R5										
18	Thiofentanyl	0.0702	57.6	0.5						
Modifications on more than one region										
19	p-Fluorobutyrfentanyl	0.657	6.2	0.06			0.908 ^b^	0.088		
20	p-Fluoroisobutyrfentanyl	0.437	9.3	0.08			1.61 ^d^	0.086	24 ^m^	0.067
21	p-Methylcyclopropylfentanyl	0.227	17.8	0.2						
22	o-Methyl-acetylfentanyl	1.508	2.7	0.02					43 ^m^	0.037
23	Ocfentanil	0.0199	203.3	1.9	0.0077 ^j^	2.5				1.0 ^q^
24	Isobutyryl-carfentanyl *	0.00319	1268.3	11.6						
25	Acetyl-α-methylfentanyl	0.349	11.6	0.11						
26	2,2′-Difluorofentanyl	0.0255	158.7	1.5			0.02 ^o^	4		
27	β-Hydroxy-3-methylfentanyl	0.0036	1123.9	10.3	0.0015 ^k^	17.3			0.18 ^i^	
28	3-Methylthiofentanyl	0.0233	173.6	1.6						
29	α-Methylthiofentanyl	0.0517	78.3	0.7						
30	β-Hydroxythiofentanyl	0.0579	69.9	0.6			1.72 ^d^	0.081	6.2 ^m^	0.26

* indicates that data for the drug in our lab have been published [8,9,10]; references mentioned in Table 3: ^a^ [12], ^b^ [13], ^c^ [14], ^d^ [15], ^e^ [16], ^f^ [17], ^g^ [18], ^h^ [19], ^i^ [20], ^j^ [21], ^k^ [22], ^l^ [23], ^m^ [24], ^n^ [9], ^o^ [25], ^p^ [26], ^q^ [27].

## Data Availability

The original contributions presented in this study are included in the article. Further inquiries can be directed to the corresponding author.
